# Recurrent Ischemic Strokes in a Young Breast Cancer Patient Due to Interatrial Septal Perforation by a Chemotherapy Port Catheter

**DOI:** 10.7759/cureus.100817

**Published:** 2026-01-05

**Authors:** Mehdi Moujahid, Mohamed Ramdani, Zouhair Lakhal, Aatif Benyass

**Affiliations:** 1 Cardiology, Mohammed V Military Instruction Hospital, Rabat, MAR; 2 Thoracic Surgery, Mohammed V Military Instruction Hospital, Rabat, MAR

**Keywords:** cardioembolic stroke, catheter malposition, implantable port catheter (pac), interatrial septal perforation, transthoracic echocardiography

## Abstract

Totally implantable venous access ports (PACs) are widely used in oncology and are generally considered safe. However, rare mechanical complications may lead to severe consequences. We report the case of a 27-year-old woman undergoing chemotherapy for breast cancer who developed recurrent ischemic strokes likely due to interatrial septal perforation by a PAC catheter. Transthoracic echocardiography revealed migration of the catheter into the left atrium, creating a direct pathway for systemic embolization. Early recognition and catheter removal prevented further events, and no recurrent ischemic events were observed during the three months of follow-up. This case highlights the importance of assessing catheter malposition when new neurological symptoms arise after PAC placement.

## Introduction

Totally implantable venous access devices (TIVADs), also referred to as port-a-catheters (PACs), are widely used in oncology patients to facilitate long-term administration of chemotherapy, parenteral nutrition, and supportive treatments. These devices are generally considered safe and effective; however, their use is not free of complications. Mechanical, infectious, and thrombotic events have been well described in the literature and may occur during insertion or throughout the lifespan of the catheter [1,2].

Catheter malposition represents an uncommon but potentially serious complication of central venous access devices. While most malpositions involve adjacent venous structures, intracardiac migration or abnormal positioning within the cardiac chambers is rare [3]. In particular, left atrial malposition is exceptional and may occur through unrecognized vascular anomalies or intracardiac septal defects, or as a result of catheter-related perforation [4].

Intracardiac catheter malposition may lead to severe complications, including arrhythmias, thrombus formation, systemic embolization, and ischemic stroke. Early recognition is therefore crucial, and echocardiography plays a key role in identifying abnormal catheter position and related complications [3,4]. Reports of ischemic stroke due to left atrial malposition of a port-a-cath catheter remain exceedingly rare, particularly in young patients without vascular risk factors.

## Case presentation

A 27-year-old woman with recently diagnosed breast cancer underwent insertion of a totally implantable venous access port via the right internal jugular vein for chemotherapy administration. The procedure was uneventful. She had no prior history of cerebrovascular disease, cardiovascular risk factors, or known thrombophilia. Over the subsequent weeks, she experienced three episodes of acute ischemic stroke, each presenting with transient focal neurological deficits.

Timeline

On Day 0, chemotherapy port implantation was done. The first ischemic event occurred approximately three weeks after port implantation, followed by two recurrent events over the subsequent four weeks.

**Figure 1:**
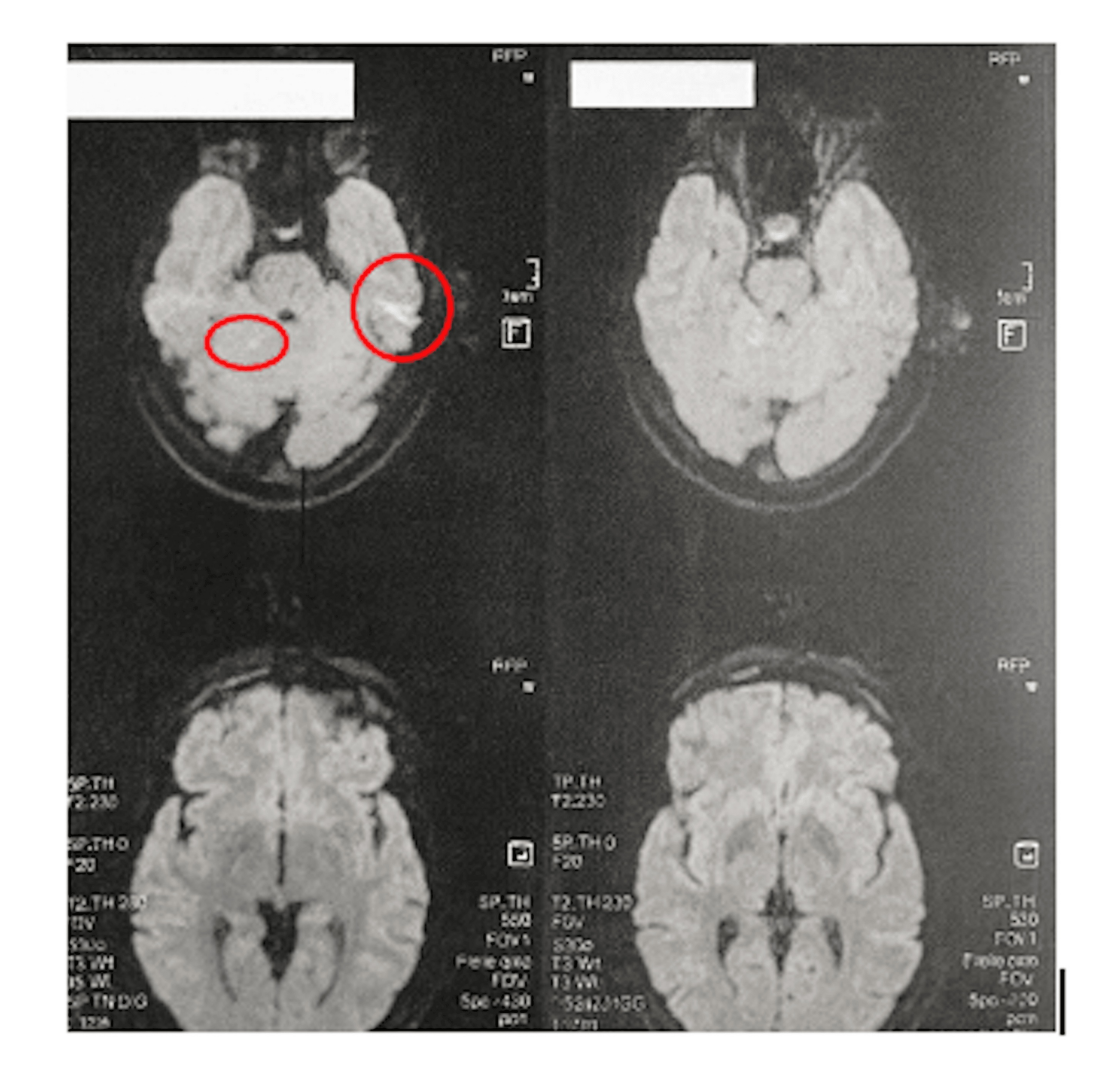
Brain MRI (diffusion-weighted imaging) showing punctate hyperintense lesions consistent with acute cerebral microinfarcts (red circles).

Brain magnetic resonance imaging (MRI) with diffusion-weighted imaging demonstrated multiple punctate hyperintense lesions involving different vascular territories, consistent with acute cerebral microinfarctions of embolic origin (Figure 1).

**Figure 2:**
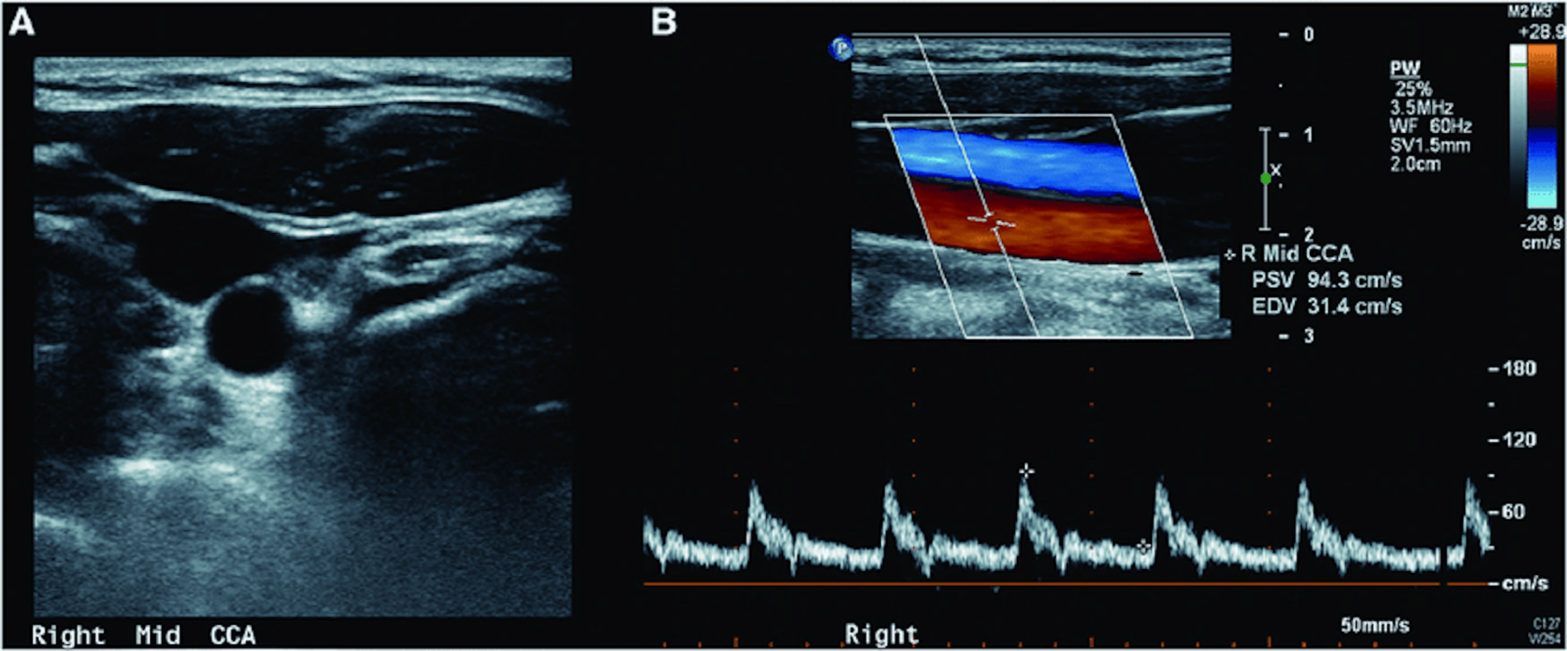
Carotid Doppler ultrasound of the right common carotid artery. The image shows normal arterial morphology on B-mode imaging (A) and normal flow characteristics on color and spectral doppler (B), with preserved laminar flow, normal peak systolic and end-diastolic velocities, and no evidence of atherosclerotic plaque or hemodynamically significant stenosis.

A comprehensive etiological evaluation for recurrent ischemic stroke was subsequently performed. Carotid Doppler ultrasound demonstrated no hemodynamically significant extracranial carotid artery stenosis (Figure 2).

Routine laboratory investigations, including complete blood count, renal and liver function tests, inflammatory markers, coagulation profile, thrombophilia screening, and antiphospholipid syndrome testing, were within normal limits. A summary of the etiological investigations is provided in Table 1.

**Table 1:** Summary of etiological investigations performed in our patient for recurrent ischemic stroke.

Etiological categories	Investigations	Results
Large artery disease	Carotid Doppler ultrasound	No hemodynamically significant extracranial carotid stenosis
Cardiac rhythm	Electrocardiogram and Holter monitoring	No atrial fibrillation detected
Hypercoagulability/thrombophilia	Protein C, Protein S, Antithrombin III; Factor V Leiden; Prothrombin G20210A	No deficiency; mutations negative
Antiphospholipid syndrome	Lupus anticoagulant; anticardiolipin (IgG/IgM); anti-β2-glycoprotein I (IgG/IgM)	Negative
Inflammation/systemic disease	C-reactive protein and erythrocyte sedimentation rate	Within normal limits

**Figure 3:**
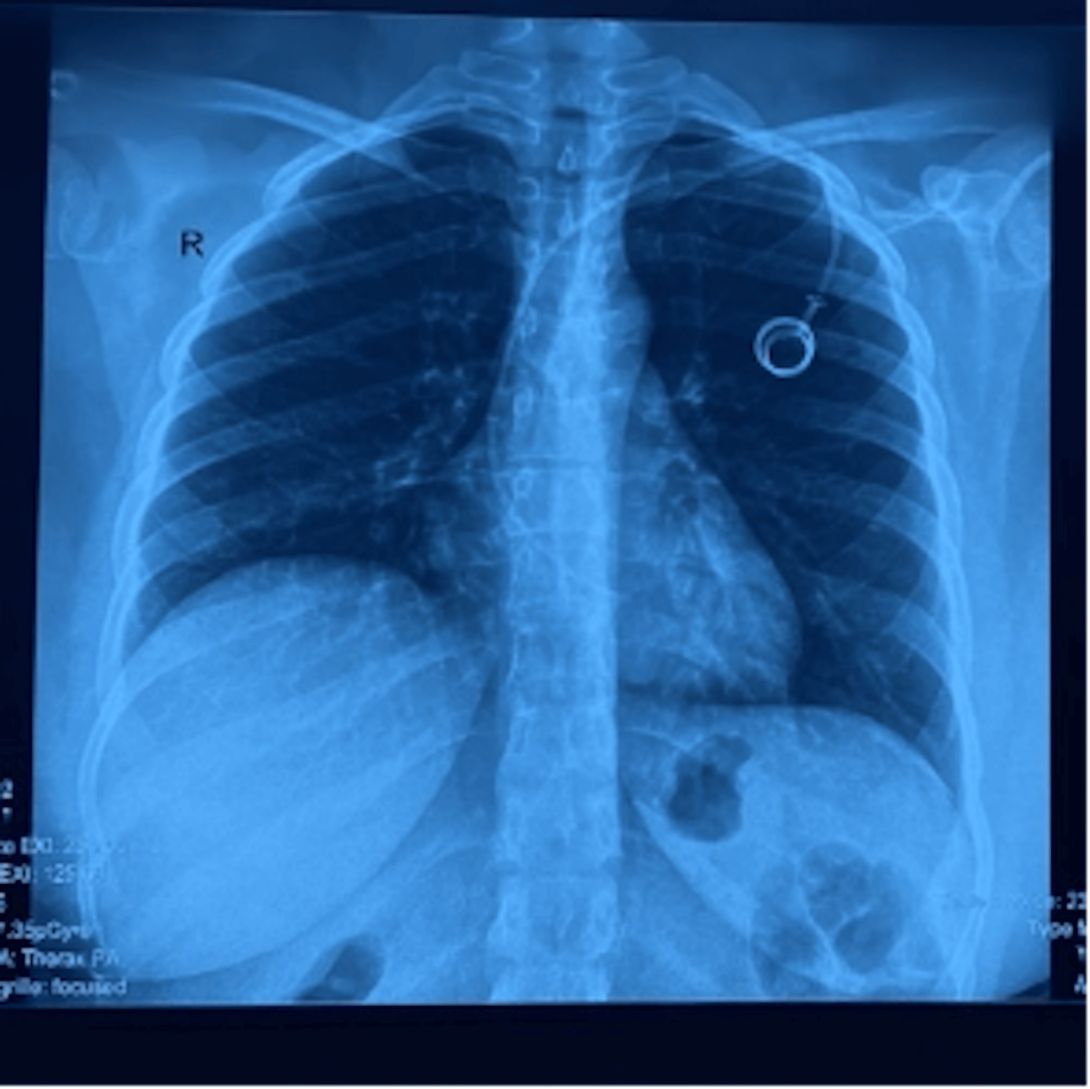
Chest X-ray showing the implantable port chamber and the course of the chemotherapy catheter.

Chest radiography confirmed the presence of the implantable port chamber and demonstrated the course of the chemotherapy catheter (Figure 3). In the absence of an alternative embolic source, cardiac imaging was pursued.

**Figure 4:**
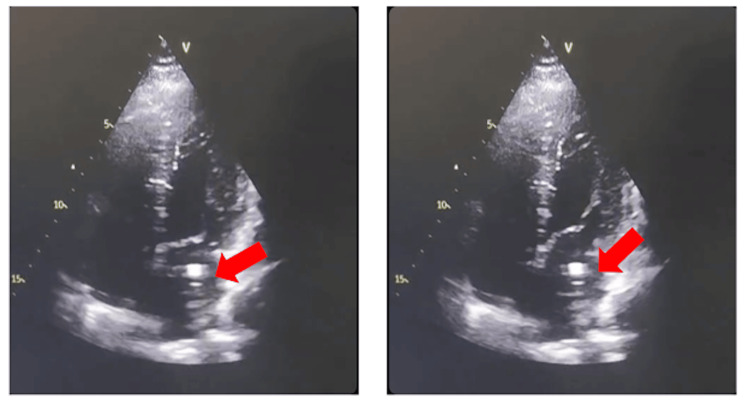
Apical four-chamber view showing the chemotherapy port catheter positioned within the left atrium (red arrows).

**Figure 5:**
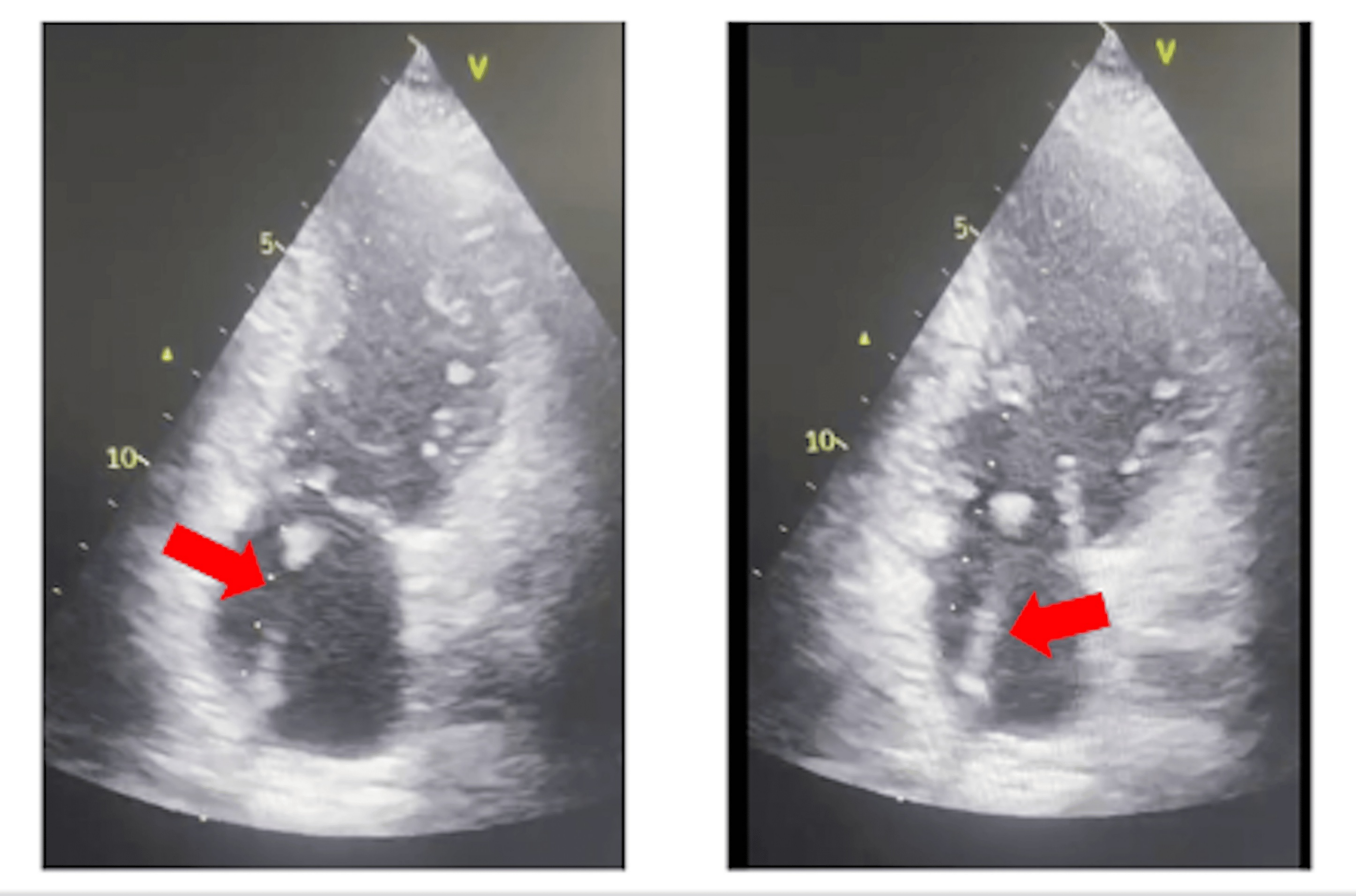
Two-chamber view showing the catheter positioned within the left atrium (red arrows).

Transthoracic echocardiography (TTE) performed on admission for the latest neurologic event revealed abnormal positioning of the port catheter, with traversal of the interatrial septum and entry into the left atrium. The catheter tip was freely mobile within the left atrial cavity, without evidence of vegetation or intracardiac thrombus (Figures 4, 5). Given adequate visualization on TTE, transesophageal echocardiography (TEE) was not required in this case. The port catheter was removed within 48 hours of echocardiographic diagnosis.

A multidisciplinary team recommended urgent catheter removal and initiation of therapeutic anticoagulation. Therapeutic anticoagulation with rivaroxaban 20 mg once daily was initiated and continued for one month after catheter removal, given the patient’s ongoing malignancy and perceived high thromboembolic risk (individualized multidisciplinary decision). No recurrent ischemic events were observed during three months of follow-up.

## Discussion

Mechanisms of interatrial septal perforation with catheter extension into the left atrium

Complications of venous access ports include thrombosis, catheter fracture, migration, and malposition [5,6]. Although catheter malposition is a recognized complication, penetration of the interatrial septum with extension into the left atrium is exceptionally rare and represents a likely iatrogenic pathway for systemic embolization.

Several mechanisms may contribute to this complication. Excessive catheter length and misdirection during insertion may result in an abnormal catheter trajectory [6,7]. In addition, repetitive cardiac motion may promote progressive erosion of intracardiac structures when the catheter tip is positioned close to the interatrial septum [5,8]. Structural vulnerability or thinning of the interatrial septum may further facilitate septal perforation.

Stroke pathophysiology

Once the catheter enters the left atrium, it may act as a nidus for thrombus formation and provide direct access to the systemic circulation, thereby increasing the risk of embolic events such as ischemic stroke [5,8].

Diagnostic role of echocardiography

Echocardiography plays a central role in the diagnosis of catheter-related complications. While transesophageal echocardiography is often considered the reference imaging modality for detecting intracardiac catheter malposition, transthoracic echocardiography may be sufficient when catheter visualization is optimal [6].

Management implications

Management typically involves prompt catheter removal, assessment for intracardiac thrombus, and anticoagulation when clinically indicated, in accordance with previously reported experiences [5,8]. Anticoagulation may be considered to reduce catheter-associated thrombus formation (foreign-body surface) and to account for cancer-related hypercoagulability, based on an individualized multidisciplinary assessment. In our patient, early catheter removal and short-term anticoagulation were associated with the absence of recurrent ischemic events during follow-up.

Limitations

Only a limited number of case reports have described ischemic stroke in patients with malposition or intracardiac migration of totally implantable venous access devices. These reports consistently highlight delayed recognition of catheter malposition as a major contributing factor to embolic complications. Our case further emphasizes the importance of maintaining a high index of suspicion for iatrogenic causes of stroke in young patients with indwelling venous access ports and negative conventional etiological investigations [9,10].

A limitation of this case is the inability to definitively differentiate catheter-induced septal perforation from passage through a previously unrecognized patent foramen ovale (PFO). However, the temporal relationship between port implantation and recurrent embolic strokes, together with echocardiographic confirmation of left atrial catheter position, strongly supports an iatrogenic mechanism.

Finally, pulmonary vein thrombosis could not be excluded because dedicated pulmonary vein imaging was not performed. Reports of ischemic stroke due to left atrial migration of PACs remain exceedingly rare, particularly in young patients without vascular risk factors.

## Conclusions

In oncology patients with recent total implantable venous access port (PAC) placement, otherwise unexplained ischemic stroke should prompt consideration of catheter malposition as a potential contributing mechanism. When acoustic windows are favorable, transthoracic echocardiography may be sufficient to identify intracardiac malposition. Although causality cannot be established definitively in a single case, the clinical course and imaging findings in our patient are consistent with catheter-related systemic embolization, and timely catheter removal may help reduce the risk of recurrent embolic events. Reports of ischemic stroke due to left atrial migration of PACs remain exceedingly rare, particularly in young patients without vascular risk factors.
